# UnFATE: A Comprehensive Probe Set and Bioinformatics Pipeline for Phylogeny Reconstruction and Multilocus Barcoding of Filamentous Ascomycetes (Ascomycota, Pezizomycotina)

**DOI:** 10.1093/sysbio/syaf011

**Published:** 2025-02-15

**Authors:** Claudio G Ametrano, Jacob Jensen, H Thorsten Lumbsch, Felix Grewe

**Affiliations:** Grainger Bioinformatics Center and Negaunee Integrative Research Center, Science and Education, Field Museum of Natural History, 1400 S DuSable Lake Shore Drive, Chicago, IL 60605, USA; Department of Life Sciences, University of Trieste, via Giorgieri 10, 34127 Trieste, Italy; Grainger Bioinformatics Center and Negaunee Integrative Research Center, Science and Education, Field Museum of Natural History, 1400 S DuSable Lake Shore Drive, Chicago, IL 60605, USA; Grainger Bioinformatics Center and Negaunee Integrative Research Center, Science and Education, Field Museum of Natural History, 1400 S DuSable Lake Shore Drive, Chicago, IL 60605, USA; Grainger Bioinformatics Center and Negaunee Integrative Research Center, Science and Education, Field Museum of Natural History, 1400 S DuSable Lake Shore Drive, Chicago, IL 60605, USA

**Keywords:** Bait set, targeted capture, phylogenomic, multilocus barcoding, next-generation sequencing

## Abstract

The subphylum Pezizomycotina (filamentous ascomycetes) is the largest clade within Ascomycota. Despite the importance of this group of fungi, our understanding of their evolution is still limited due to insufficient taxon sampling. Although next-generation sequencing technology allows us to obtain complete genomes for phylogenetic analyses, generating complete genomes of fungal species can be challenging, especially when fungi occur in symbiotic relationships or when the DNA of rare herbarium specimens is degraded or contaminated. Additionally, assembly, annotation, and gene extraction of whole-genome sequencing (WGS) data require bioinformatics skills and computational power, resulting in a substantial data burden. To overcome these obstacles, we designed a universal target enrichment probe set to reconstruct the phylogenetic relationships of filamentous ascomycetes at different phylogenetic levels. From a pool of single-copy orthologous genes extracted from available Pezizomycotina genomes, we identified the smallest subset of genetic markers that can reliably reconstruct a robust phylogeny. We used a clustering approach to identify a sequence set that could provide an optimal trade-off between potential missing data and probe set cost. We incorporated this probe set into a user-friendly wrapper script named Universal Filamentous Ascomycete Target Enrichment (UnFATE) (https://github.com/claudioametrano/UnFATE) that allows phylogenomic inferences without requiring expert bioinformatics knowledge. In addition to phylogenetic results, the software provides a powerful multilocus alternative to Internal transcribed spacer (ITS) based barcoding. Phylogeny and barcoding approaches can be complemented by an integrated, pre-processed, and periodically updated database of all publicly available Pezizomycotina genomes. The UnFATE pipeline, using the 195 selected marker genes, consistently performed well across various phylogenetic depths, generating trees consistent with the reference phylogenomic inferences. The topological distance between the reference trees from literature and the best tree produced by UnFATE ranged between 0.10 and 0.14 (normalized Robinson-Foulds) for phylogenies from family to subphylum level. We also tested the *in vitro* success of the universal baits set in a target capture approach on 25 herbarium specimens from 10 representative classes in Pezizomycotina, which recovered a topology congruent with recent phylogenomic inferences for this group of fungi. The discriminating power of our gene set was also assessed by the multilocus barcoding approach, which outperformed the barcoding approach based on ITS. With these tools, we aim to provide a framework for a collaborative approach to build robust, conclusive phylogenies of this important fungal clade.

Filamentous ascomycetes (subphylum Pezizomycotina) represent most of the diversity in the largest phylum of Fungi Ascomycota and is comprised of species that adopt one of the widest ranges of lifestyles in the fungal kingdom. Many of these species have relevance in medicine, biotechnology, and food production, while many others have pathogenic potential. Given the importance of these species, it is surprising that the Ascomycota tree still needs to be fully resolved. Although many major conflicts were solved recently using genome-scale data (Shen et al. 2020), phylogenomic analyses often suffer from incomplete taxon sampling. For instance, 50% of the described Ascomycota orders have no representative genome assembly (Hill et al. 2021). Even when the phylogenetic inference is conducted using a few well-established genetic markers, the accuracy of the results can be compromised by incomplete taxon sampling. For instance, the estimated number of taxa for which the commonly used rDNA large subunit locus (LSU or 28S) is available on National Center for Biotechnology Information (NCBI) Genbank is about 20,000 (data not shown), which roughly corresponds to the number of species that were accepted for the Dothideomycetes class alone 10 years ago (Kirk et al. 2008; Schoch et al. 2009). Within the last 10 years, many more species have been described, since a relevant portion of the fungal diversity is still unknown (Hawksworth and Lücking 2017).

Shotgun genome sequencing (Venter et al. 1998) based on high-throughput sequencing (Ronaghi 2001) technologies provided the necessary impulse for a framework shift in phylogenetic studies. It is now a common practice to consider the evolutionary information carried by hundreds to thousands of genomic loci. Given the large amount of genomic data that is used for phylogenetic work, studies are often coherently labeled as phylogenomic studies (Philippe et al. 2005). However, the term was first introduced by Eisen (1998) to highlight the need to include evolutionary relationships to infer gene function better. Following this trend, new bioinformatic tools to reconstruct phylogenomic trees emerged and former phylogenetic programs were updated to handle phylogenomic-level data sets (Zhang et al. 2018; Kozlov et al. 2019; Minh et al. 2020). However, even with the possibility of obtaining high-quality fungal genomes from high-throughput sequencing (Rhoads and Au 2015; Jain et al. 2016), sequencing and assembling a draft fungal genome in good quality still presents some challenges. For example, symbiotic organisms and microfungi from complex matrices, such as soil, need to be either isolated or sequenced as metagenomes, and herbarium samples often have poor DNA quality, which is unsuitable for WGS applications.

The economic burden and technical challenges in identifying fungal samples or producing a robust phylogeny of the clade of interest using WGS can be avoided using reduced representation methods. Reduced representation methods require less sequencing and can offer pre-designed pipelines to analyze the sequence data, which can otherwise be overwhelming when hundreds of samples are sequenced (Good 2012). These methods can be based on restriction enzyme-selected loci (e.g., Restriction site associated DNA sequencing, RADseq) or are based on specific loci to be targeted and captured (e.g., ultra-conserved elements, Faircloth et al. 2012). Restriction-based approaches are only effective if restriction sites are conserved among taxa (e.g., RADseq, Davey and Blaxter 2010). Therefore, methods, such as RADseq are better suited to infer shallow evolutionary relationships and are often used for population genomic approaches (Grewe et al. 2017, 2018; Salas-Lizana and Oono 2018). However, target capture methods can obtain complete coding sequences (Schott et al. 2017), which allows the selection of specific marker genes of interest based on their function or their phylogenetic performance. Hence, targeted capture allows the reconstruction of phylogenies within deeper time scales. In addition, it can access an additional source of data from off-target reads, which have been exploited to assemble organellar markers and nuclear rDNA (Weitemier et al. 2014; Allio et al. 2020). Among the possible approaches to enrich a particular set of loci of interest (target enrichment), hybrid capture using nano beads has recently become the technical solution of choice (Mamanova et al. 2010; Mertes et al. 2011). Target capturing and the enrichment of specific loci for a specific taxon has especially proven beneficial when working with older herbarium specimens with degraded DNA, or when symbiotic organisms are targeted (Brewer et al. 2019; Forrest et al. 2019; Grewe et al. 2020). Although phylogenetic studies using this technique have the additional cost of the enrichment step, advanced multiplexing techniques and other strategies (Glenn et al. 2019; Hale et al. 2020) can help dilute this cost. In addition, the marker selection and the probe design process can be both time-consuming and bioinformatically challenging. Bait sets usually are designed from 1 or a few reference sequences (Widhelm et al. 2019), and the performance of these baits rapidly decreases when the reference sequence diverges too far from the targeted DNA (Liu et al. 2019). To overcome these limitations, universal bait sets have been developed, such as for angiosperms (Johnson et al. 2019) and flagellate plants (Breinholt et al. 2021).

The opportunity to sequence multiple genes with target capture allows a multilocus barcoding approach. Multilocus barcoding (Blaxter 2004) has already proven to be a powerful tool, overcoming the limitations of single-locus barcode approaches based on rDNA (Mallo and Posada 2016). In addition, Liu et al. (2017) showed that a multilocus approach based on tens to hundreds of nuclear protein-coding loci outperformed the Cytochrome c Oxidase I (COI) barcode routinely used for fish when sister species were tested. The standard barcode for Fungi is rDNA ITS (Beimforde et al. 2014). However, this marker lacks the needed resolving power in several groups of Fungi (Roe et al. 2010; Pino-Bodas et al. 2013; Lücking et al. 2020). Thus, we established a multilocus approach to barcoding, to enhance the possibility of reliably describing fungal diversity.

The recent increase in the popularity of target enrichment sequencing led to the development of many bioinformatics tools. Existing bioinformatics pipelines allow users to both identify suitable loci and to design probes (Mayer et al. 2016; Campana 2018; Chafin et al. 2018); other pipelines allow the processing of data from target enrichment sequencing (Johnson et al. 2016; Andermann et al. 2018), whereas others allow reconstructing phylogenomic trees from hundreds to thousands of loci applying various approaches to data filtering and tree reconstruction (Tice et al. 2021; Resl et al. 2022). However, no existing solution can simultaneously accomplish all these steps, for all filamentous ascomycetes.

We aimed to fill this gap by building a comprehensive and user-friendly pipeline that integrates pre-designed universal baits for filamentous ascomycetes and automates all required steps of bioinformatic processing from the raw data to the phylogenetic tree. The pre-designed universal bait sequences will be accessible to the public as well as the computational pipeline Universal Filamentous Ascomycete Target Enrichment (UnFATE) bait set and wrapper script which provides a 1-click straightforward phylogenomic workflow. The universal baits set and UnFATE pipeline, together with the existing genomic resources, will help to populate a database of marker sequences for filamentous ascomycetes. This database will provide a framework for a collaborative approach to build robust phylogenies of filamentous ascomycetes, similar to what the Angiosperms353 (Johnson et al. 2019) and GoFlag 451 (Breinholt et al. 2021) projects are accomplishing in flowering and the flagellate plants tree of life, respectively (Baker et al. 2021; Maurin et al. 2021; McDonnell et al. 2021). This database of filamentous ascomycete genes allowed the implementation of a multigene barcoding approach. Consequently, a barcoding option that takes advantage of all NCBI publicly available filamentous ascomycete genomes is already implemented in the first version of UnFATE.

## Materials and methods

### Gene Selection and Bait Set Design

Initially, we determined the minimum number of genes that can produce a reliable phylogeny of Pezizomycotina. We used a pilot data set of 162 genome assemblies from NCBI assembly (11 December 2019; Supplementary Table S1), picking 1 assembly per genus and excluding assemblies with <90% of BUSCO completeness, >10% of duplicated BUSCOs (BUSCO 5.4.2). From these assemblies, we selected the widely used universal single-copy orthologs (BUSCO) (Simão et al. 2015) as a starting pool gene set for Ascomycota (1706 protein-coding genes, Orthodb v10, Waterhouse et al. 2013). The ortholog genes that were retrieved were then aligned with mafft using parameters -maxiterate 1000 and -localpair (Katoh and Standley 2013) and subsequently block-filtered with Gblocks using relaxed parameters (Castresana 2000 ). Genes with aligned exons shorter than 600 bp were discarded. To identify the minimum number of genes that were required to receive a well-supported, stable phylogeny, a set of 20 genes were stepwise added to a concatenated matrix until the full gene set was used. Of each matrix with 20 to all genes, we calculated a maximum likelihood tree with IQTREE 2.1.2 (Minh et al. 2020). This procedure was repeated a second time, but the genes were ordered by increasing substitution rates as done previously (Grewe et al. 2020). The rates were calculated using PAML 4.9 baseml (Yang 2007) using a calibration point of 449 Mya for the Pezizomycotina root (average value among Beimforde et al. 2014; Nelsen et al. 2020 and; Prieto and Wedin 2013). For the constrained tree for the rates analyses, we used the single-locus tree inferred by reconstruct single-locus ML trees (RaxML) 8.2 (Stamatakis 2014), rooted with the Orbiliomycetes-Pezizomycetes clade. In addition to calculating species trees from concatenated matrices, we used a coalescent-based approach with Accurate Species TRee ALgorithm (ASTRAL) (Mirarab et al. 2014) to reconstruct species trees from a stepwise increasing number of single loci RAxML trees. Differences in the topology between the produced trees were measured by normalized Robinson-Foulds distance (nRF) implemented in the ETE toolkit 3.1.3 (Huerta-Cepas et al. 2016). A nRF distance matrix was built to assess the topological distances between all reconstructed phylogenies and identify at which gene number the phylogenetic trees were not further improving. In addition to nRF, all phylogenetic trees were used to calculate the average bootstrap support values as another parameter to compare the quality of the resulting phylogenetic trees.

After assessing the topological stability and average support values variation produced by a progressively larger number of loci using the pilot data set, a bigger data set was built to identify suitable genes in an amount consistent with the findings of the preliminary analyses. All sequenced draft genomes of Pezizomycotina available at NCBI on 6 May 2020 were processed with BUSCO5.4.2 (Benchmarking single-copy orthologs, Simão et al. 2015). The most complete assembly for each species was then retained in a comprehensive data set. BUSCO genes from this data set were aligned with mafft 7.52 and block-filtered with Gblocks 0.91. The genes still retaining at least 600 bp of aligned exons were used for the k-medoids clustering to identify the best representative sequences for each gene, that is, the lowest number of sequences in an alignment that represented all known Pezizomycotina species (throughout the remaining text, these alignments of reference sequences from few representative species are referred to “medoid alignments.”). K-medoid clustering works like the k-means approach, except it picks an actual value (i.e., a sequence) from the alignment instead of using the mean value. The aim of this method is to select the sequences (medoids) that minimize the distance to the surrounding values. This approach is described in Johnson et al. (2019) and was used to build a universal bait set for angiosperms. We tested maximum allowed distances from medoids of 0.20, 0.25, 0.30, and a maximum number of medoids of up to 70. We used 100 iterations for each medoid number tested since the clustering method starts from a random sequence in the alignment on each iteration. The genes that successfully converged, were then represented by a number of representative sequences smaller or equal to the maximum, which allowed at least 95% of the sequences in the alignment to be within the maximum distance selected. The clustering parameters (maximum distance from the medoid, medoid number allowed) and the minimum length of exons were adjusted to obtain at least the minimum number of genes that were optimal for topological and nodal support of phylogenetic trees (see above).

The alignments of the selected genes were used to RAxML. Single-locus topologies were used to build a nRF distance matrix, which was used to calculate the average distance of any single-locus tree from the others and the species tree. This distribution of average distances was used to discard outlier topologies using the Thompson-tau test. The single-locus trees distance matrix was then used as an input for a 2D Multi-Dimensional Scaling and DBscan clustering (scikit-learn 1.0, Pedregosa et al. 2011). The clustering method was used to evaluate the presence of topology clusters corresponding to possible alternative discordant evolutionary histories.

Unfiltered “medoid alignments” were realigned in a codon-aware fashion with MACSE2 (Eddy 2009; Ranwez et al. 2018). From these alignments, HMMER 3.3.2 profiles were created with hmmbuild and these profiles were used to retrieve the target genes from selected transcriptomes and *Aspergillus nidulans* annotated genome (Supplementary Table S2) using hmmsearch (Finn et al. 2011). These alignments were then used to manually curate the “medoid alignments” that were then used to design the bait set. This additional step allowed us to delete introns when possible, and to check the ends of the coding sequences predicted by Augustus (Stanke et al. 2006).

The manually curated “medoid alignments” were sent to Arbor Biosciences (Ann Arbor, MI) to produce the RNA baits set. The complete baits set comprises of 80 bp baits and is designed in a 2× tiling fashion. Ns in consecutive runs of 1–10 were changed to Ts. As a control, all baits were BLAST (2.14.1) searched in 13 reference genomes. All baits that produced multiple hits, those with more than 35% repeats, or those in a region with more than 35% repeats were discarded. Baits with extreme GC content were also discarded (25% < GC < 67%). These baits sequences will be made available to the public for reproduction.

### Pipeline Workflow

**Figure 1.**
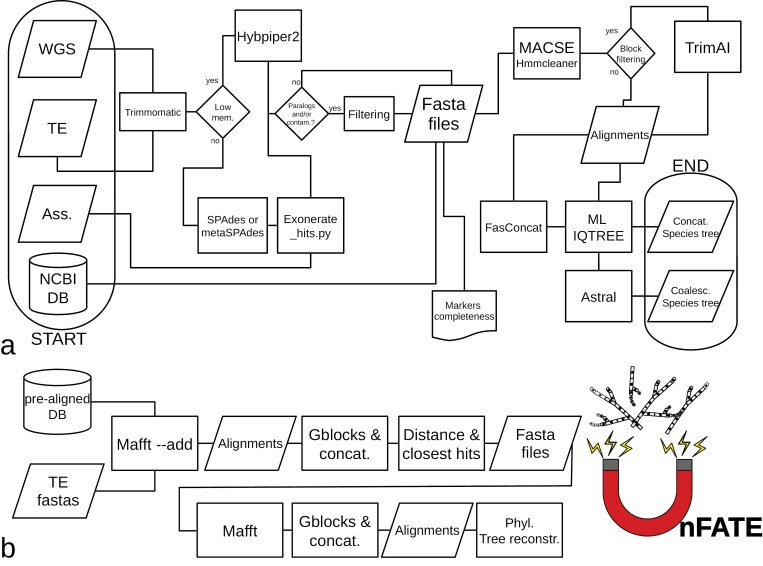
(a) Workflow of the UnFATE pipeline; (b) Workflow of UnFATE in its multilocus barcode version. Parallelograms are used for inputs/outputs, rectangles for processes, diamonds for decisions, cylinders for databases. TE: Target Enrichment; WGS: Whole-Genome Sequencing; Ass.: Assembly; DB: Database; ML: Maximum likelihood.

We implemented a user-friendly wrapper script called UnFATE (github.com/claudioametrano/UnFATE). The software allows the processing of filamentous ascomycete target enrichment data and the processing of data from other sources, such as genome assemblies or raw sequence data. As shown in the workflow (Fig. 1a), target enrichment data are trimmed by Trimmomatic (Bolger et al. 2014) before they are assembled with metaSPAdes 3.15.5 (default; Nurk et al. 2017) or Hybpiper 2.1.8 (-y option in UnFATE: mapping to reference, then SPAdes; Johnson et al. 2016). Raw WGS data are trimmed by Trimmomatic 0.39 before they are either assembled with SPAdes 3.15.5 (default; Bankevich et al. 2012) or Hybpiper (see above). SPAdes and MetaSPAdes assemblies are then processed using a script from the Hybpiper workflow (exonerate_hits.py), which exploits Exonerate 2.4.0 (Saari et al. 2017) for appropriate gene extraction. After gene retrieval from the data sources, it is highly recommended to apply the filtering step (-s argument), especially for samples from unknown targets. When this filtering is applied, genes that are retrieved in multiple copies (2 or more) in a sample and do not meet the coverage depth condition are removed. By default, a copy must be at least 10 times deeper covered than any other copy obtained. If the optional filtering step is not used, the copy that satisfies the depth condition or, if this fails, the most similar copy to the reference sequence, according to the Exonerate alignment, is retained. It is worth noting that the Hybpiper2 default approach, while is suitable in many cases where contamination is unlikely (e.g., plant contaminants for a plant sample), is not ideal for fresh or herbarium fungal samples, which may contain a significant amount of other fungi efficiently captured—especially when a universal bait set is used. The completeness of the extracted genes in fasta file format is evaluated by comparing them with the reference sequence length. A heatmap and a.csv table are produced to indicate the level of completeness of each gene (provided in the “fastas” output folder). In the next step of the pipeline, nucleotide, and amino-acid translations of the extracted genes are codon-aware aligned with the OMM_MACSE 10.02 pipeline (Ranwez et al. 2018; Di Franco et al. 2019). This pipeline includes a segment-based filtering step based on Hmmcleaner, which is performed in addition to an optional blocks filtering approach using TrimAl 1.4 (Capella-Gutiérrez et al. 2009). Amino-acid and nucleotide alignments are then used both for concatenated (FASconCAT-g 1.04, Kück and Longo 2014) ML species tree, and for single-locus ML tree inferences (both using IQTREE 2.1.1 and selecting the best substitution model with Model Finder Plus) followed by species tree inference using ASTRAL 5.7.7.

### Universal Bait Set and Pipeline Testing

Including the data specifically generated to test the universal baits set (see below), here are the data sets that were used to evaluate the performance of the pipeline, the selected genes, and the bait set:

1) Twenty-five herbarium specimens of variable age (2004–2023, see NCBI SRA metadata, Data Availbility section) from 10 major classes in Pezizomycotina, specifically selected, DNA extracted, captured with the universal baits, and sequenced as 2 × 150 bp reads on the Illumina Novaseq6000, to test the 195 genes and the universal bait set; 2) High and low (<15×) coverage WGS data from Ametrano et al. (2022) and Pizarro et al. (2018), respectively (195 UnFATE genes); 3) high-quality target enrichment data capturing a wide phylogenetic range of Lobariaceae (96 samples) and other Peltigerales (35 samples) by baits (400 genes) designed from *Lobaria pulmonaria and Evernia prunastri* (Widhelm et al. 2019, 2021); and 4) several phylogenomic data sets from literature; from shallower to deeper: 93 genomes in Aspergillaceae (Steenwyk et al. 2019), 48 lichen metagenomes in Parmeliaceae (Pizarro et al. 2018), 101 genomes in Dothideomycetes (Haridas et al. 2020) and the Pezizomycotina subset of the fungal kingdom phylogeny (Li et al. 2021), containing 758 genomes (195 UnFATE genes).

The performance of the bait set and the pipeline was assessed as follows:

1) The completeness of the loci retrieved by UnFATE was estimated both in terms of loci completeness (in comparison to the length of the reference gene) and of percentage of loci retrieved (presence/absence) using WGS data (Pizarro et al. 2018; Ametrano et al. 2022), Peltigerales target enrichment data (Widhelm et al. 2019, 2021) and the newly sequenced Pezizomycotina specimens using the universal bait set; 2) The impact of mapping and coverage threshold parameters in Hybpiper on the completeness of the retrieved genes were also assessed with the Peltigerales target enrichment data; we relaxed the default BLAST e-value (10^-10^) to 10^-7^ and 10^-3^ and the default coverage threshold (8×) to 3× and 1×. We kept the sequence identity threshold for the Exonerate alignment to a default value of 55% for both Hybpiper and “assembly first” methods; and 3) the performance of our 195 phylogenetic markers and the pipeline was assessed in term of topological consistency using the 4 data sets mentioned earlier (Pizarro et al. 2018; Steenwyk et al. 2019; Haridas et al. 2020; Li et al. 2021). Phylogenies produced by the UnFATE wrapper and the originally published trees were compared both in terms of overall topological distance (nRF) and in detail assessing the topological differences and the nodal support. In addition to the ultrafast bootstrap nodal support provided by IQTREE2 and the local posterior probability from ASTRAL, the level of conflict of single gene topologies was summarized by Phyparts (Smith et al. 2015) and phypartspiecharts.py (github.com/mossmatters/phyloscripts/tree/master/phypartspiecharts).

The Ascomycota BUSCO gene set we used as the starting point for marker selection contains genes that are rarely duplicated. However, duplication, or other events, such as ploidy or contamination (especially when herbarium or fresh environmental specimens instead of cultures are used), can cause more than 1 copy to be obtained from target enrichment, or (meta)genomic data. Therefore the amount of gene copies was assessed using the paralog_retriever.py script from Hybpiper, both in 400 genes from Lobariaceae and in the newly generated sequences of the 195 genes in Pezizomycotina.

### Pre-computed Pezizomycotina Database

To reduce the computational burden, a pre-computed database containing the selected gene set from any assembly available on NCBI assembly portal (29 July 2021) can be queried within UnFATE (NCBI DB, Fig. 1, option -n). The user can choose any main taxonomic rank from NCBI taxonomy belonging to the subphylum Pezizomycotina. The samples belonging to the selected rank will then be automatically added to the alignment produced by the UnFATE pipeline without the need for the time-consuming processing of assemblies or raw reads archives.

The database was built with the following steps: 1) the assembly reports for the assemblies belonging to kingdom Fungi were downloaded with ncbi-genome-download (https://github.com/kblin/ncbi-genome-download) and filtered to retain only accession numbers, 2) the taxonomy was associated to each accession (https://github.com/claudioametrano/get_taxonomy_with_edirect), fixed to retain only the main ranks (class, order, family, genus, and species), and only samples classified at least to the genus level, 3) the accessions belonging to Pezizomycotina were downloaded with ncbi-genome-download, and 4) UnFATE was used to mine for the Pezizomycotina gene sets from the genomes and to build comprehensive fasta files. The database composed of the taxonomy file and the alignments can be queried through the UnFATE command line. The nucleotide version of the database was also prealigned with mafft (Katoh and Standley 2013) which speeds up the multilocus barcode approach of UnFATE.

### Multilocus Barcode Mode and “-n AUTO” Mode

multilocus barcoding was added to the pipeline as an additional mode in UnFATE to provide fast multigene barcoding identification results, without running a complete phylogenetic analysis. The method follows the outline of Liu et al. (2017) that exploits the speed of a distance method coupled with the accuracy of a phylogenetic reconstruction method to infer the sister clade taxa of the query sample. The multilocus barcode mode (barcode_wrap.py, Fig. 1b) starts from a prealigned version of the Pezizomycotina database in its nucleotide version. The newly sequenced fasta file produced by UnFATE is added to these alignments using mafft (--add). The alignments are then block-filtered and concatenated. The polished alignments are used to calculate the pairwise distance (Biopython Bio.Phylo.TreeConstruction module, DistanceCalculator, “blastn” scores) between the query sequence and each sequence in the database to determine the closest sequences. The twenty most similar sequences are selected (with up to 4 sequences belonging to the same species). The query sequence and the most similar sequences are then realigned with mafft, block-filtered, and concatenated to produce a more specific alignment. Finally, a ML phylogenetic tree (IQTREE2) is generated from the concatenated DNA alignment which resolves the sister taxon/a relationship of the queried sequence. This distance-based method used for the barcode mode can also be used in the UnFATE main_wrap.py automatic mode (-n AUTO) to automatically select closely related sequences to the samples to be included in the phylogeny. The final tree includes the samples to be investigated, plus at most twenty additional tips for each sample. The barcode mode runs 1 sample at a time and does not allow to filter out multiple-copy genes; the user can use the “-n AUTO” mode of main_wrap.py together with the “-s” argument, to both barcode multiple samples simultaneously and to apply the filtering.

The effectiveness of the distance method used for the barcoding mode to discriminate between species using an increasing amount of genetic data was assessed by random sampling (5 replicates) an increasing number of loci (3–150), and comparing the intraspecific and interspecific distances of multiple individuals (3 to 5 individuals of each species; https://github.com/jacobnjensen1/UnFATE_paper_scripts). The effectiveness of the barcoding method, and the barcoding potential of our genes, were assessed by comparing the identification results from our approach to the result obtained by ITS barcoding using BLAST (Altschul et al. 1990). For this approach, the complete assembly database was mined for the ITS region by ITSx (Bengtsson-Palme et al. 2013) after fragmenting the longest contigs to fragments of 100 Kb (ITSx maximum sequence length). An ITS BLAST database containing the same species as our genome-based database was then built. When no ITS region was found in the genome assembly, the ITS database was manually completed by sequences of the same species (where possible, the same strain) from the NCBI nucleotide database to ensure a fair comparison of the 2 barcoding methods.

## Results

### Gene Selection and Bait Design

The topology and support values of phylogenetic trees calculated from data

sets of increasing gene numbers plateaued rapidly, indicating that about 200 genes are suitable to reconstruct the Pezizomycotina phylogeny with a resolution similar to all BUSCO genes. For randomly sampled 200 genes, the nRF distance between sampled genes and the reference was 0.06 for concatenated and 0.01 for coalescence-based species tree inferences. For 200 genes that were ordered by substitution rate, the nRF distance was 0.06 for concatenated and 0.04 for coalescence-based tree inferences (Supplementary Figs. S1–S4). Moreover, the average support value (bootstrap) for the concatenated ML trees produced with 200 genes was 99.8 for randomly sampled genes and 95.5 when slow to fast-evolving genes were used. The average support value (posterior probability) for coalescence-based trees was 0.981 for randomly sampled genes and 0.977 when slow to fast-evolving genes were used (Supplementary Fig. S5 and S6). Therefore, about 200 genes were set as the minimum number of genes that can construct reliable phylogenies. This number of genes achieved the best balance between the required phylogenetic accuracy (phylogenetic signal increases with the number of genes) and bait set size (more genes will result in a bigger bait set) to represent the entire filamentous ascomycetes phylogeny effectively.

We used the complete set of 1706 semi-universal BUSCO orthologs as a starting point to select about 200 genes. According to the original publication, these universal single-copy orthologs should be present in 90% of the species and they are rarely duplicated (Simão et al. 2015). The alignments used to retrieve the representative sequences by k-medoids clustering were built from 3124 Pezizomycotina draft genome assemblies. Of these genomes, 1163 remained (Supplementary Table S3) after filtering the most complete for each species based on the BUSCO completeness benchmark. The k-medoids clustering converged for 210 genes, represented by up to 50 medoids per gene, with 95% of the sequences within a distance of 0.25 from their medoid (the representative sequence). Of these 210 genes, ten genes were discarded because they were identified as outliers in the distribution of single-locus topology average nRF distance; 2 additional genes were removed by the same method, as they performed consistently poorly in test runs using phylogenomic data sets from the literature. An additional 3 genes were removed because they were rich in repetitive regions (RGG boxes, Thandapani et al. 2013). The DBscan clustering approach performed on the nRF distance matrix from single-locus topologies did not identify any dominant alternative topology cluster (Supplementary Fig. S7). The final gene set consisted of 195 genes represented by 3109 individual sequences for a total length of 4,748,098 bp. The bait set for target capture approaches was designed from these sequences in a 2× tiling design. The baits comprised of 100,000 80 bp RNA baits, which survived the filtering steps (see methods).

### Pipeline Testing: Average Length, Fraction of Genes and Genes in Multiple Copies

Under the UnFATE pipeline, WGS data can be processed either with Hybpiper, which uses reads mapping or by assembling the WGS data with SPAdes, which does not require read mapping (“assembly first,” default). The pipeline tests indicated that the 2 methods provide similar completeness for high coverage WGS DATA (Hybpiper: 97.1%, “Assembly first”: 96.7%; Supplementary Table S4). Hybpiper had the advantage of reduced memory footprint since it only assembles reads that map onto the reference sequences. This is due to Hybpiper using a mapping approach (e-value 10^-10^ using BLASTx, if an amino-acid reference file is provided) before assembling, and a coverage threshold for the assembled contigs (by default equal to 8). However, the “assembly first” method, on average, reduced the amount of missing data (average marker length completeness: Hybpiper 70.4%, “Assembly first” 96.6%; Supplementary Table S4), when low coverage WGS data was used (Grewe et al. 2020) or, when reference sequences are divergent enough from target DNA (see below).

We also assessed the performance of the pipeline using high quality 96 samples in Lobariaceae captured by baits designed from 2 taxa (*Lobaria pulmonaria* and *Evernia prunastri*) (Widhelm et al. 2019), and testing the universal bait set we designed, sequencing 25 herbarium specimens from ten major classes in Pezizomycotina (Supplementary Table S5). In the Peltigerales data set, we determined that using Hybpiper2 or “assembly first” retrieved loci which were on average 0.86 (st. dev. 0.19) and 0.93 (st. dev. 0.16) the length of the respective reference sequences. The “assembly first” approach provided, on average, 8% more loci than Hybpiper2. Relaxing the Hybpiper2 parameters (e-value, coverage) provided an improvement of 4% more loci. It is worth noting that the reference sequence can strongly influence the completeness of the gene retrieved. The difference between the 2 methods, in terms of loci retrieved, became indeed larger when the sample diverged from the 1 used to design the bait set; Supplementary Table S6 shows, using the Widhelm et. al (2021) Peltigerales data, that just 5%–6% loci retrieved increase for Lobarioideae (*Lobaria* was used as reference sequences indeed), but up to 36% more loci are retrieved, using “assembly first,” in more distantly related clades, such as Coccocarpiaceae. In the Pezizomycotina data set, obtained using the universal bait set, we determined that using Hybpiper2 or “assembly first” retrieved loci which were on average 0.92 (st. dev. 0.06) and 0.97 (st. dev. 0.04) the length of the respective reference sequences. The “assembly first” approach provided on average 1% more loci than Hybpiper2. In terms of the number of copies, capture using a bait set specifically designed on 1 to few taxa (Widhelm et al. 2019) resulted in a low fraction of multiple-copy genes (~4%) mostly in 2 copies (average 2.07–2.11 copies in multiple-copy genes), while the universal bait set retrieved a relevant portion of multiple-copy genes (31% with on average 3.4–3.6 copies; Supplementary Table S5).

### Pipeline Testing: Comparison Between the 195 UnFATE Genes Phylogenies and Previously Published Phylogenomics Trees

We compared phylogenies of different depths produced with UnFATE based on 195 universal filamentous ascomycete genes with the respective reference phylogenies from the literature. The published maximum likelihood (ML) genus-level phylogeny of *Aspergillus* and *Penicillium* (DNA, concatenation, 1668 loci) (Steenwyk et al. 2019) compared to the ML phylogeny by UnFATE with a nRF distance of 0.11. The distances produced by all other topologies from the original study to the UnFATE topologies had a nRF range of 0.10–0.18. (Supplementary Fig. S8a). The original phylogenies were only marginally influenced by the data set used (nucleotide or AA) and the different reconstruction methods (ML or coalescent-based), while UnFATE topologies were slightly less consistent. The comparison (Supplementary Fig. S9) highlighted only a minor and 2 major incongruences: The sister relationship of *Aspergillus steynii*, the placement of *Aspergillus ochraceoroseus* within the genus *Aspergillus*, and the placement of the genera *Monascus* and *Xeromyces*.

The comparison of the *Parmeliaceae* family-level phylogeny from Pizarro et al. (2018) (2256 loci) showed mostly consistent topologies with 1 major incongruence. The ML phylogenies based on a concatenated data set had a nRF distance of 0.13 in relation to the published tree. The 2D-MDS summarizes the distances between the 4 topologies produced by UnFATE and the 2 topologies from the original study (Supplementary Fig. S8b, nRF range 0.13–0.27). The plot highlights that the UnFATE topologies cluster according to the reconstruction method used, with the UnFATE coalescent-based species trees as having the furthest topologies. The comparison of topologies (Supplementary Fig. S10) highlighted 2 minor and 1 major incongruence: the placement of the *Usnea*-*Cornicularia* clade, the sister relationship of *Arctoparmelia centrifuga* and *Pseudevernia furfuracea*, and the placement of *Relicina intertexta*, which is not supported in the UnFATE phylogeny.

The comparison between the Dothideomycetes class-level phylogeny from Haridas et al. (2020) (aa, concatenated, ML, 738 loci) and the UnFATE tree (DNA, concatenated, ML, 195 loci) had a nRF distance of 0.14 (the nRF range between the UnFATE and the original phylogenies was 0.09–0.20) (Supplementary Fig. S8c). The comparison of topologies (Supplementary Fig. S11) shows few incongruences, mostly in the placement of samples belonging to small orders, such as Patellariales, Acrospermales, Lineolatales and *incertae sedis* taxa such as *Coniosporium*, or within Pleosporales (*Aaosphaeria, Melanomma, Pleomassaria*, and *Massariosphaeria*).

The comparison at the highest taxonomic level measured the distance between the phylogenies produced by Li et al. (2021), pruned to the taxa that belong to Pezizomycotina (from a fungal kingdom tree), and the UnFATE phylogenies. The nRF distance between the published tree (290 loci aa concatenated, ML) and the UnFATE ML concatenated DNA tree (195 loci) was 0.11. The nRF distance between the UnFATE topologies and original phylogenies ranged between 0.08 and 0.20 with the UnFATE topologies mostly clustering by data type (DNA, aa) and the Li et al. (2021) one mostly clustering by reconstruction method (concatenation, coalescent-based) (Supplementary Fig. S8d). In this case, the most distantly related UnFATE topology was built from aa alignments and a coalescent-based approach. The comparison of topologies (Supplementary Fig. S12) shows some major incongruences in various lineages involving: *Cryomyces*, Venturiales, Thermoascaceae, *Aspergillus* (few species), Sordariales-Coniochaetales, Erysiphaceae, Diaporthales, Glomerellales, Sarocladiaceae, Stachybotryaceae, Bionectriaceae*, Trichothecium*, Ophiocordycipitaceae, and Cordycipitaceae.

The ASTRAL species trees reconstructed from single-locus ML trees of the universal target genes highlighted a high degree of conflict between gene trees and species trees at every level of phylogenetic depth (Supplementary Figs. S13–S16). The pie charts show a high number of nodes having a prevalence of topologies discordant from the ASTRAL species tree. The nodes with a high level of conflict are characterized by multiple conflicting topologies—none of the nodes in any of the 4 test phylogenies was characterized by a prevalence of 2 leading topologies. Most nodes show full support because the published phylogenomic trees are based on many more loci than the reduced representation method we implemented with UnFATE (except for the full fungal kingdom phylogeny from Li et al. 2021). The UnFATE phylogenies present a few more nodes than the references, which are not fully supported, considering the phylogenies up to the class level. However, all the incongruences highlighted by the tanglegrams (Supplementary Figs. S9–S12), except the *Relicina intertexa*, were fully supported. The UnFATE Pezizomycotina tree, being a deeper and larger phylogeny, presents the highest number of unsupported bipartitions, but often these clades also corresponded to incongruences with the reference phylogeny. Relevant examples include: Venturiaceae (bootstrap value = 4), Thermoascaceae (82), Erysiphaceae (4), Sordariales-Coniochaetales (84) Sarocladiaceae (2), Stachybotryaceae (2), Ophiocordycipitaceae (2), and Cordycipitaceae (2).

### Pipeline Testing: A Proof of Concept Pezizomycotina Phylogeny Using the Universal Bait Set

We extracted DNA from 25 herbarium samples, followed by target capturing and sequencing using our universal bait set to assess its efficacy. An average of ~4.8 × 10^6^ (st. dev. 2.4 × 10^6^) 2 × 150 bp high quality reads per sample were produced (phred score of raw reads > 28); of these an average of 37% (st.dev 16%) were on target.

The resulting data set was complemented by 1 sample per class from publicly available genomes. As the fraction of multiple-copy genes was higher using our universal bait set in comparison to more specific ones (Supplementary Table S5), and the herbarium samples being of heterogeneous quality, biomass and age, we ran the UnFATE pipeline both with and without the “strict filtering” argument (-s), which deletes every gene in multiple copies, except those for which 1 copy has at least n times the coverage of the others (default 10×).

**Figure 2.**
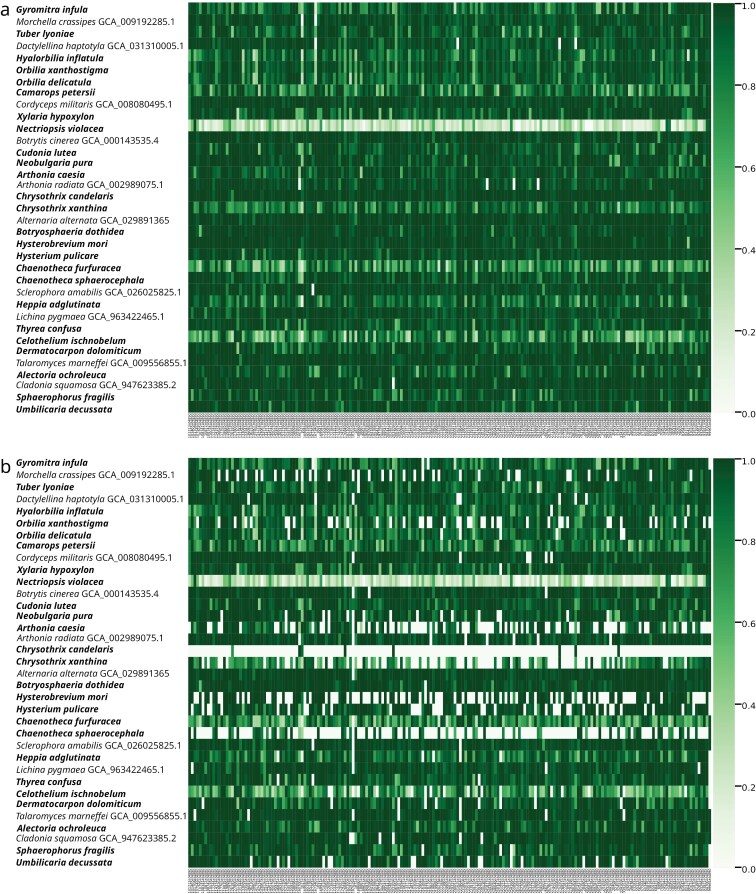
Completeness of the 195 UnFATE genes (horizontal axis) as a fraction of the reference sequence lengths for each of the 25 target captured herbarium samples (in bold) and the 10 reference genome (vertical axis) when: (a) all the copies are considered, or (b) only the genes found in single copy, or in multiple copy with a copy having at least ten times the average coverage of any other copy (-s argument). Samples are ordered according to the phylogenetic placement (at the class level) and in alphabetical order within the class.

The heatmap (Fig. 2) shows that this strict filtering increased the number of missing loci of the target captured herbarium samples from nearly zero (only 1 gene in 1 sample completely missing; Fig. 2a) to about 12% of missing loci (Fig. 2b). This increase in missing data due to strict filtering, along with the average number of copies for multiple-copy genes being higher than 2 (Supplementary Table S5), highlights that the herbarium samples might have more contamination from other fungi rather than an abundance of paralogous genes (which should be in 2 copies characterized by similar coverage). Filtering also increased the number of missing loci in the ten high-quality assembly samples, but to a much lesser extent. Since these assemblies were derived from whole-genome sequencing projects, it is likely that duplicated gene copies represent paralogs, as they were mostly found in 2 copies with similar coverage. Consequently, due to 31% of loci being in multiple copies, 12% of loci were missing in target enrichment herbarium samples. Similarly, for the 4% of genes found in multiple copies, a comparable proportion of missing loci was observed in the assemblies. Two herbarium samples were discarded: *Chrysothrix candelaris*, for the very high fraction of multiple-copy genes not solved by coverage, which left the sample with only 8 genes in single copy, and *Nectriopsis violacea*, which mostly produced short markers due to poor DNA quality. Other samples with a relevant amount of missing genes were retained to assess how robust the method was to missing data. After alignment and block filtering, the DNA data matrix consisted of ~256,000 bp, on average ~1300 bp per locus, and ~86,000 amino acids, on average ~441 amino per locus.

**Figure 3.**
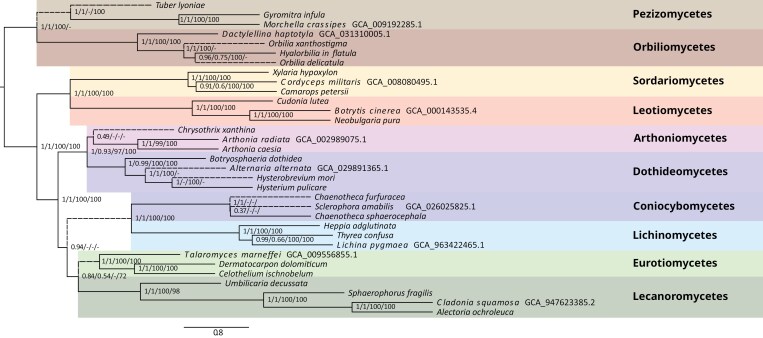
Coalescence-based (ASTRAL) species tree of ML single-locus trees (nucleotides). Support values are near branches (posterior probability from this phylogeny/bootstraps of the coalescence-based species tree of amino-acid ML trees/ultrafast bootstraps from the ML tree from concatenated nucleotide alignments/bootstraps from the ML tree from concatenated amino-acid alignments). No support value (“-”) and dashed branch indicate an incongruent topology between this phylogeny and at least one of the other 3 inferred phylogenies (coalescence aa, concatenated nt, concatenated aa). Tips with NCBI accession numbers reported are genome assemblies, while the rest of the tips are taxa from targeted capture.

The phylogenies inferred by default by the UnFATE pipeline are 4: 2 summary species trees (DNA and AA) and 2 ML phylogenies based on concatenated alignments (see methods section). The most consistent phylogenies were obtained by the summary method (Fig. 3) which produced 2 phylogenies (DNA and AA) of 0.17 nRF (normalized Robinson-Fould distance). The same input data used for the concatenation approach produced 2 phylogenies 0.33 nRF far apart, similar to the distance of the phylogenies obtained with both a different inference method (summary/concatenation) and data (DNA/AA) (0.30 nRF). The phylogenies were mostly consistent in basal nodes with few incongruences. The most relevant was the placement of the sister clades Lichinomycetes/Coniocybomycetes (recently merged under the name Lichinomycetes, together with other classes not considered here, Díaz-Escandón et al. 2022) in relationship with the Eurotiomycetes/Lecanoromycetes clade. The number of genes used (up to 195), intermediate between a few-loci and a phylogenomic analysis, allowed the support values not to saturate, both in the case of the summary method and the concatenation, highlighting few critical nodes of the presented phylogeny, which did not receive full support.

### Multilocus Barcoding

**Figure 4.**
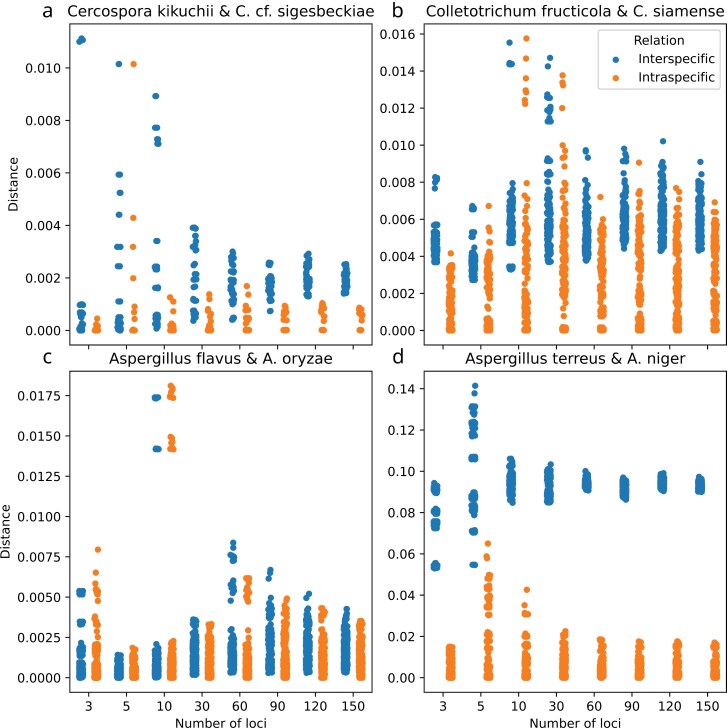
Intra- (orange) and inter- (blue) specific distance between multiple samples of 4 pairs of closely related species. Distances were calculated using alignments of an increasing number of randomly sampled markers among the UnFATE genes. Normalized distance is shown on vertical axis, number of sampled loci on horizontal axis.

Multilocus barcoding was implemented in the UnFATE pipeline and provides a fully automated feature that allows barcoding of selected taxa based on the database integrated into UnFATE. The comparison of intra- and inter-species distances using randomly sampled loci highlighted a generalized increase in the resolution power of the method with an increasing number of loci (Fig. 4). The UnFATE barcoding mode uses a distance method as a fast-skimming approach to select the closest sequences from the database. These sequences are subsequently used to reconstruct a small phylogeny (20 samples, up to 4 samples per species, in default mode) and to infer the most reliable sister clade of the queried sample. The species we selected to test the power of the distance method exemplify 4 possible scenarios: 1) Few genes can lack the resolving power to discriminate closely related species, such as *Cercosposa kikichii* and *C.* cf. *sigesbeckiae.* However, a higher number of genes both increased the precision (smaller variance among replicates and individuals) and the discrimination accuracy (interspecific distance > intraspecific distance) of the barcoding (Fig. 4a). 2) Another possible scenario is when distributions were not completely disjointed, but different (Fig. 4b). 3) In a third scenario, species that share most of the genomic sequence, such as *Aspergillus flavus* and *A. oryzae*, and which could be reliably discriminated only by complete genome SNP analysis (Chang 2019) were not resolvable (Fig. 4c). 4) Taxa which are not considered sister species were resolved even with few genes, but a higher number of loci still positively impacted the precision of the analysis (Fig. 4d).

A comparison of the UnFATE multilocus barcode and the ITS BLASTn barcode approach was also applied (Table 1), using the same 4 species couples’ scenarios from above. In addition, we used 2 more species, that is, *Colletotrichum acutatum* C71 and *Fusarium fujikuroi* IMI 58289, which belong to taxa that were difficult to barcode previously (Lücking et al. 2020). ITS BLAST barcode on *Aspergillus flavus* AF31 (Supplementary Table S6) provided a large number of equally good matches, belonging to multiple species names: *A. minisclerotigenes, A. tritici, A. flavus, A. luchuensis, A. oryzae*, and *A. parasiticus.* In comparison, the UnFATE multilocus barcoding (Supplementary Fig. S14) detected the queried samples clearly as *A. flavus* by placing it in a monophyletic clade. Furthermore, *Aspergillus terreus* ATCC20542 ITS sequence blasted with high similarity only on *A. terreus* sequences (Supplementary Table S7), and the multilocus approach placed the query sample in a monophyletic *A. terreus* clade (Supplementary Fig. S15). These results are in accordance with the high interspecific distance of the species to closely related species (Fig. 4). *Colletotrichum acutatum* C71 ITS blasted with high similarity to sequences from *C. scovillei, C. acutatum*, and *C. fioriniae* which made an identification ambiguous (Supplementary Table S8). The UnFATE approach clustered the sample with itself (Supplementary Fig. S16). Still, the other selected sample of *C. acutatum* was instead spread within the phylogeny, confirming the difficulty in distinguishing well circumscribed species in the *C. acutatum* species complex. *Cercospora* cf. *sigesbeckiae* PP 2012 003 ITS blasted with similarity to sequences belonging to *C. kikuchii, C*. cf. *sigesbeckiae* and *C*. cf. *flagellaris* which complicates an identification with a single barcode (Supplementary Table S9). However, identification was possible in the multilocus tree which placed the queried sequence in a monophyletic clade of *C*. cf. *sigesbeckiae*, sister to *C. kikichii.* The ITS sequence of *Colletotrichum fructicola* Nara gc5 (Supplementary Fig S17) blasted correctly with high similarity to *C. fructicola* sequences, except for a *C. gloeosporioides* sequence (Supplementary Table S10). The multilocus approach correctly identified the queried sample as *C. fructicola* placing it in a clade exclusively composed of *C. fructicola* samples (Supplementary Fig. S18), except for the same (possibly misidentified) *C. gloeosporioides* sample present in the ITS-based barcoding. *Fusarium fujikuroi* IMI5828 ITS blasted with a perfect similarity score closer to many other *F*. species, such as *F. proliferatum, F. mangiferae, F. globosum, F. fujikuroi*, and *F. annulatum*, therefore it was not possible to barcode it correctly only using ITS (Supplementary Table S20). However, the multilocus phylogeny correctly identified the query sample as *F. fujikuroi* (Supplementary Fig. S19).

**Table 1.** Comparison between ITS BLASTn and multilocus UnFATE barcode, a tick is assigned if only the species that corresponds to the query sequence obtained the best alignment score (for ITS BLASTn barcode) or if the query species is delimited in a monophyletic clade (for UnFATE multilocus barcoding), if not a cross is assigned

	ITS BLASTn	UnFATE barcoding
Aspergillus flavus F13	✘	✔
Aspergillus terreus ATCC20542	✔	✔
Colletotrichum acutatum C71	✘	✘
Cercospora cf. sigesbeckiae	✘	✔
Colletotrichum fructicola Nara gc5	✔	✔
Fusarium fujikuroi IMI58289	✘	✔

## Discussion

This study focuses on developing a universal set of single-copy orthologous genes to enhance phylogenomic analyses of Pezizomycotina. The set of 195 genes developed in this study delivered reliable phylogenetic inferences, comparable in quality to inferences that used thousands of loci. The UnFATE pipeline and its integrated database provide a new bioinformatic tool, that will help accelerate the production and analysis of phylogenomic data sets from various sources, including target enrichment, whole-genome sequencing, and assemblies. In addition to phylogenetic reconstructions, a multilocus barcode approach of UnFATE provides a new tool, which can be used to assign species names in problematic taxa when single-locus barcoding has insufficient species-level resolving power. Furthermore, the universal gene set eliminates the need to sequence thousands of markers, which can be challenging when processing hundreds to thousands of samples. Sequencing genomes with low coverage and skimming for the markers of interest is also an option, but it produces unnecessary data and a higher computational burden. While UnFATE can perform genome skimming, it can be avoided for projects aiming primarily to reconstruct phylogenetic relationships. A more efficient approach is targeted capture, coupled with multiplexing using advanced indexing methods (e.g., Glenn et al. 2019), which can produce hundreds of markers from potentially thousands samples at high coverage even on a small bench-top Illumina sequencer.

The clustering method and the filtering steps applied to get the final 195 gene set, starting from the widely used BUSCO genes, allowed a reduced representation approach, identifying loci whose phylogenetic performance was evaluated by comparisons with high-quality phylogenies on a wide range of phylogenetic depth. The comparisons between UnFATE results and reference published phylogenies highlighted the effectiveness of the gene set at different evolutionary scales. The topological distances from the reference trees were similar for all the tested data sets (0.10–0.14 nRF).

Despite an overall excellent similarity of UnFATE phylogenies to previously published phylogenies, a small number of incongruences were discovered. The Aspergillaceae phylogeny from Steenwyk et al. (2019) is mostly focused on the genera *Aspergillus* and *Penicillum*, however, 2 samples from the *Monascus* and *Xeromyces* genera in the UnFATE trees are either basal to the whole *Penicilium*-*Asprergillus* clade (Steenwyk et al. 2019) or basal only to *Aspergillus* (UnFATE DNA ML tree). In both positions, these taxa maintain their sister relationship. However, the results change in UnFATE phylogenies based on amino-acid alignments where these taxa are placed in accordance with the published phylogeny. However, also in the original phylogenomic inference of Steenwyk et al. (2019) these alternative placements of the 2 genera were proposed.

The Parmeliaceae phylogenies also differed in the placement of a few samples. *Relicina intertexta* is the most striking one, compared to the previously published tree by Pizarro et al. (2018) and Grewe et al. (2020). However, the position in the UnFATE concatenated phylogeny lacks support, and the taxon is placed congruently in the UnFATE ASTRAL phylogeny. The UnFATE placement of the Usneoid clade as a sister to all remaining Parmeliaceae is consistent with the tree in Grewe et al. (2020). However, this clade is sister to the Anzioid clade in Pizarro et al. (2018). *Arctoparmelia centrifuga* and *Pseudevernia furfuracea* are basal to each other but switched in the UnFATE tree compared to the tree in Pizarro et al. (2018). Both taxa are in a weakly supported sister relationship in Grewe et al. (2020).

Dothideomycetes phylogenies were consistent across different taxon sampling, data types, and methods applied. The incongruences found highlight the difficulty of reconstructing some phylogenetic relationships in Dothideomycetes. The trees show similar problems to the phylogenies in Ametrano et al. (2019) and Haridas et al. (2020), which used comparable taxon samplings. For example, *Lineolata rhizophorae*, which is basal to Aulographales and Venturiales in Ametrano et al. (2019) and instead basal to the rest of Pleosporomicetidae and sister to *Patellaria atrata* in Haridas et al. (2020), is sister to *Coniosporium* (another problematic taxon) in the UnFATE phylogeny. While some of these positions are only weakly supported in Ametrano et al. (2019) and Haridas et al. (2020), the positions of these taxa have strong support in the UnFATE DNA ML phylogeny.

The comparison of Pezizomycotina phylogenies also highlighted some differences between the UnFATE phylogeny and the published tree. For example, the placement of *Cryomyces*, which is consistent in Ametrano et al. (2019), Li et al. (2021), and Shen et al. (2020), differs in the UnFATE phylogeny when DNA data is used. Interestingly, *Cryomyces* in the UnFATE DNA concatenated ML tree is reported as sister to *Coniosporium* on long terminal branches. This placement could be caused by long-branch attraction in the presence of incorrectly modeled multiple substitutions. Amino-acid alignments do not have to deal with abundant and possibly saturated synonymous substitutions, which can occur in deep phylogenies. Therefore, this taxon is placed consistently with the reference phylogenies when amino-acid alignments are used. Other similar examples are Venturiales, *Byssochlamys*, and Erysiphaceae, whose placement is congruent in all of the previously mentioned phylogenies but is consistently placed by UnFATE only when amino-acid data is used.

Differences were also detected among UnFATE phylogenies, driven by data type (DNA or aa) and reconstruction method. For example, it was highlighted by previous studies (Zhang et al. 2018, 2020; Wang et al. 2021; Widhelm et al. 2021) that coalescence-based summary species trees can have a high level of node conflict even when advanced summary reconstruction methods are used. Even though we discarded gene trees that were topological outliers to decrease conflict, other methods could be applied to filter out problematic loci with low signal-to-noise ratio, and further improve species tree reconstruction and conflict level in specific lineages. Metrics such as Partitioned Coalescence Support (Gatesy et al. 2017, 2019), or methods to collapse poorly supported node in single-locus phylogenies before summarizing them into a species tree, could help decrease the problematic nodes. This, or other similar approaches, would also possibly improve the concordance between the results obtained by concatenation and coalescence-based species trees.

The final gene set is constrained by the need to limit the size of the bait set. It is therefore composed of many conserved protein-coding genes, possibly more suitable for deeper rather than shallower phylogenies. However, we achieved a satisfactory trade-off between bait set size, gene number, and phylogenetic performance of the gene set. At larger time scales, some taxa were misplaced possibly due to sequence divergence and saturation. Therefore, the UnFATE phylogenies should be interpreted with care, using the most suitable method/data type case by case.

When the universal bait set was applied to an *in vitro* experiment to capture and sequence the 195 genes from various quality herbarium specimens, both the power of the method and some critical points, that must be taken into consideration, emerged. The bait set was able to capture at least 1 sequence from nearly every gene in every sample and it showed a fairly homogeneous behavior in capturing a wide phylogenetic range of fungi, nonetheless few samples resulted in an high amount of missing data, possibly because competing with more efficiently captured sequences (even within the sample itself). Since the bait set was designed to be as universal as possible, and the samples’ DNA did not come from pure cultures, often multiple contaminant copies were obtained. In more than half of these, coverage depth helped to identify the copy belonging to the target organism. When this was not possible, the markers were precautionary discarded in those samples, increasing the amount of missing data.

Some samples, often from smaller lineages (Arthoniomycetes, Lichinomycetes, Coniocybomycetes) with fewer genomic resources available, showed a low fraction of on-target reads, though other samples from the same lineages performed consistently better. While this is expected for such a universal probe set, additional enrichment tests using high-quality DNA from pure cultures—eliminating intra-sample competition and DNA quality issues—could help better elucidate any relevant phylogenetic bias. However, a conscious approach to sample grouping and customized hybridization conditions can mitigate this issue. The test using herbarium samples highlighted the crucial role of DNA quality and purity, particularly regarding contaminant fungi, which can be captured as efficiently as—or even more efficiently than—the target organism’s DNA, in obtaining consistent results.

If abundant paralogous genes are suspected in the data, the Hybpiper paralog_retriver can be run on any UnFATE run to assess the gene copy number, since UnFATE is based on the same folder structure of Hybpiper2 (Johnson et al. 2016). The user can then decide if the amount of missing data generated by the strict filtering in UnFATE is worth being re-assessed and loci partially reintroduced into the workflow by a paralogy resolution pipeline such as, ParaGone (Jackson et al 2023). ParaGone is designed to be compatible with Hybpiper2, and therefore also works with the UnFATE output, however, it is not integrated in the pipeline. At a stage, where UnFATE highlighted that multiple contaminant copies can affect analyses more than the missing data, we adopted a precautionary deletion approach. This is crucial when working with a universal bait set and specimens from unknown fungal species, as the only help in detecting the right copy among many is provided by the coverage. Possible real paralogs (genes in exactly 2 copies of similar, high coverage) should be rare among the orthologs we selected, as shown by the very low amount of missing data generated from assembled genomic resources. Ploidy and duplication events can potentially complicate the picture, although these fungi are usually haploid in their vegetative phase. Despite these limitations, the phylogenies produced by the bait set and the UnFATE pipeline were consistent with the currently accepted phylogenetic relationship of the main classes in Pezizomycotina.

*In silico* UnFATE runs based on genomic resources proved the concatenation as a still useful approach (Gatesy and Springer 2014; Gatesy et al. 2017), especially in the context where single loci are producing highly conflicting trees. However, as this is often the case, an advanced coalescence-based species tree method was also implemented, providing valuable insight into the data. This was especially true for the proof of concept phylogeny produced by our bait set. The phylogeny inferred by this method using nucleotide data proved to be the closest to the currently accepted relationships among the 10 major classes we selected to try our baits and phylogenetic pipeline. One major node proved to be particularly difficult to resolve, but this holds for a recent phylogenomic analysis that proposed 3 alternative topologies for the placement of Lichinomycetes and their relationship to Lecanoromycetes and Eurotiomycetes (Díaz-Escandón et al. 2022).

Our testing effort managed to find a suitable number of genes, trying to minimize missing data during the capture process (e.g., reference sequences for bait design identified by clustering) and data analysis (e.g., mapping vs “assembly first”), while maintaining phylogenetic accuracy, discarding putative paralogous genes and contaminant copies.

Most of the protein-coding genes we selected for the universal gene set lacked the sequence variability to be applied on their own as effective barcodes. However, when used collectively in a multilocus approach, they outperformed the single-locus barcode approach based on the universal barcode for Fungi (ITS). In general, although there will be cases, that will not be solved even using this 195 genes multilocus approach, such as species described using other species concepts (e.g., morphological) without genetic evidence, the same species described by different names (anamorph/teleomorph), or (putative) species only distinguished by few Single Nucleotide Polymorphisms (SNPs) across the genome (Payne et al. 2006; Han et al. 2024), this multilocus approach improves the resolutive power of the barcoding approach, as shown by the case studies we reported. However, it is worth noting that the accuracy of the barcoding results is influenced by the quality and completeness of the reference database, which is the case for any barcoding approach. Being based on publicly available genomic resources, our database is currently way less comprehensive than the ITS database from NCBI GenBank or any curated ITS database (such as UNITE, Nilsson et al. 2019). However, the difference between species with genomic resources and with classical barcodes available will decrease in the long run. With this increasing database, UnFATE and its universal gene set can effectively be applied as a multilocus barcoding approach, to reveal the identity of unknown samples or it can be used to get both a comprehensive phylogeny and the closest related samples from the UnFATE database in the same phylogenetic tree.

## Conclusions

Our baits set, the command line wrapper script and the support database provide a framework for a collaborative approach to build a robust phylogeny of Pezizomycotina at different phylogenetic levels. Phylogenomics by reduced representation could be applied routinely for systematics and integrative taxonomy and present an alternative to the classically adopted markers, or whole-genome sequencing.

This first version of UnFATE provides a fast, general workflow that can be applied by most scientists without specific bioinformatics expertise. UnFATE produces phylogenetic trees, launching a single command. The pipeline allows further customization and additional analyses by the user, as every step is described, and intermediate results are retained and organized in the output folder. The universality of the bait set allows the building of deep phylogenies but also proved to be robust for shallower phylogenies. The high resolving power of the universal gene set was also highlighted by its capability to resolve sister and closely related species, which ITS failed to resolve. Hence, we also developed the multilocus barcoding approach, which is integrated into UnFATE. As the efficacy of this approach relies on the database completeness, we aim to keep the database updated, collecting both data sets from target enrichment experiments and mining the UnFATE genes from newly sequenced genomes periodically.

In summary, our program and bait set provide a novel approach to improve filamentous ascomycetes phylogeny and systematics based on molecular data.

## Data Availability

UnFATE tool and database available at CGA GitHub page: https://github.com/claudioametrano/UnFATE. Scripts for multilocus barcoding test available at JJ GitHub page: https://github.com/jacobnjensen1/UnFATE_paper_scripts. Data generated in this study have been deposited in the NCBI Sequence Read Archive (SRA) under the BioProject ID PRJNA1216762. Data files (bait sequences, alignments, and trees) can be found in the Dryad data repository: https://doi.org/10.5061/dryad.tht76hf1x. All genomic data used to benchmark the pipeline are available at https://www.ncbi.nlm.nih.gov/assembly, accessions of the assemblies we used are reported in Supplementary Information. Target enrichment data from Grewe et al. (2020), Widhelm et al. (2019) and Widhelm et al. (2021) are available at https://www.ncbi.nlm.nih.gov/sra.

## Supplementary material

Data available from the Dryad Digital Repository: https://dx.doi.org/10.5061/dryad.tht76hf1x
